# Dicer-2 promotes mRNA activation through cytoplasmic polyadenylation

**DOI:** 10.1261/rna.065417.117

**Published:** 2018-04

**Authors:** Olga Coll, Tanit Guitart, Ana Villalba, Catherine Papin, Martine Simonelig, Fátima Gebauer

**Affiliations:** 1 Gene Regulation, Stem Cells and Cancer Programme, Centre for Genomic Regulation (CRG), The Barcelona Institute of Science and Technology, 08003-Barcelona, Spain; 2 Universitat Pompeu Fabra (UPF), 08003-Barcelona, Spain; 3 Institute of Human Genetics, CNRS UMR9002-University of Montpellier, mRNA Regulation and Development, 34396-Montpellier, France

**Keywords:** Dicer-2, Wispy, translation, cytoplasmic polyadenylation

## Abstract

Cytoplasmic polyadenylation is a widespread mechanism to regulate mRNA translation. In vertebrates, this process requires two sequence elements in target 3′ UTRs: the U-rich cytoplasmic polyadenylation element and the AAUAAA hexanucleotide. In *Drosophila melanogaster*, cytoplasmic polyadenylation of *Toll* mRNA occurs independently of these canonical elements and requires a machinery that remains to be characterized. Here we identify Dicer-2 as a component of this machinery. Dicer-2, a factor previously involved in RNA interference (RNAi), interacts with the cytoplasmic poly(A) polymerase Wispy. Depletion of Dicer-2 from polyadenylation-competent embryo extracts and analysis of *wispy* mutants indicate that both factors are necessary for polyadenylation and translation of *Toll* mRNA. We further identify *r2d2* mRNA, encoding a Dicer-2 partner in RNAi, as a Dicer-2 polyadenylation target. Our results uncover a novel function of Dicer-2 in activation of mRNA translation through cytoplasmic polyadenylation.

## INTRODUCTION

Dynamic regulation of the mRNA poly(A) tail length allows rapid and accurate control of gene expression in time and space. In oocytes and embryos, a long poly(A) tail promotes translation, while poly(A) tail shortening beyond a certain threshold leads to mRNA decapping and degradation (Zhang et al. 2010; Eckmann et al. 2011; Subtelny et al. 2014). Cytoplasmic poly(A) tail elongation stimulates mRNA translation and stability in a wide variety of biological situations, including oocyte maturation, early embryonic development, neuronal plasticity, cell proliferation and senescence, cell identity, inflammation, metabolism, circadian gene expression and hibernation (Kojima et al. 2012; Weill et al. 2012; Ivshina et al. 2014; Elewa et al. 2015; Grabek et al. 2015).

The biochemistry of cytoplasmic polyadenylation has been deciphered in vertebrates, where two sequences in the 3′ UTR of substrate mRNAs are required: the U-rich cytoplasmic polyadenylation element (CPE) and the A(A/U)UAAA polyadenylation hexanucleotide (Hex). The CPE is recognized by the CPE-binding protein (CPEB, a family of four members) while the Hex is bound by the multisubunit cleavage and polyadenylation specificity factor (CPSF). Together with the scaffolding protein Symplekin, these factors recruit the cytoplasmic poly(A) polymerase GLD-2 to elongate the adenosine tail at the 3′ end of the mRNA (for review, see Villalba et al. 2011; Weill et al. 2012; Ivshina et al. 2014). The poly(A) tail is then recognized by poly(A)-binding protein (PABP), which contacts the cap-binding complex at the 5′ end of the mRNA to stimulate translation initiation.

The cytoplasmic polyadenylation machinery is conserved in *Drosophila melanogaster*. During late oogenesis in this organism, the CPEB1 homolog Orb and the Wispy poly(A) polymerase—which belongs to the GLD-2 family—function to promote the polyadenylation of several mRNAs (Chang et al. 1999; Benoit et al. 2008; Cui et al. 2008, 2013; Norvell et al. 2015). Wispy also polyadenylates a large number of maternal mRNAs during the first 2 h of embryogenesis, a time that concurs with little or no transcription (Benoit et al. 2008; Cui et al. 2008, 2013; Eichhorn et al. 2016; Lim et al. 2016). During these early stages of development, there is no expression of Orb, and a weak expression of the CPEB2-4 homolog Orb2 is detected, suggesting that CPEB-independent mechanisms of cytoplasmic polyadenylation might ensue (Vardy and Orr-Weaver 2007; Rangan et al. 2009; Hafer et al. 2011). Indeed, the polyadenylation of the transcript encoding the morphogen and innate immunity effector Toll occurs independently of the CPE and Hex (Coll et al. 2010). Importantly, titration experiments showed that polyadenylation of *Toll* is not competed by RNAs containing functional CPEs and Hex, indicating that an alternative, noncanonical machinery is responsible for polyadenylation of this transcript (Coll et al. 2010). A fragment of *Toll* 3′ UTR located after the stop codon (termed “polyadenylation region” or PR) retained polyadenylation capacity and was able to efficiently compete polyadenylation of *Toll*, suggesting that relevant factors bind to this region.

Here we identify Dicer-2 as a component of the noncanonical machinery that binds to PR. Initial experiments showed that Wispy, but not Orb2 or Symplekin, binds to *Toll* mRNA and functions in *Toll* polyadenylation. Wispy lacks RNA-binding domains and is thought to associate to substrates via RNA-binding factors. To identify these factors, we used RNA affinity chromatography using PR as bait. Surprisingly, the RNA interference (RNAi) factor Dicer-2 bound to PR together with Wispy. Dicer-2 interacts with the carboxy-terminal domain of Wispy, containing the nucleotidyl transferase domain. Squelching and depletion experiments indicated that Dicer-2 promotes cytoplasmic polyadenylation and translation of *Toll*. This function is independent of the RNAi machinery because (i) Ago-2, a partner of Dicer-2 in the RNAi machinery, does not bind to PR, and (ii) Dicer-2 interacts with Wispy in a complex that lacks the RNAi factor R2D2. In addition to *Toll*, other cytoplasmic polyadenylation substrates bind to Dicer-2. Among these, we identify *r2d2* mRNA as a novel substrate regulated by Dicer-2 in vitro and in vivo, suggesting the existence of a positive loop to reinforce RNAi in the *Drosophila* early embryo. Our results reveal a novel function of Dicer-2 in cytoplasmic polyadenylation.

## RESULTS

### Wispy is involved in cytoplasmic polyadenylation of *Toll* mRNA

**FIGURE 1.**
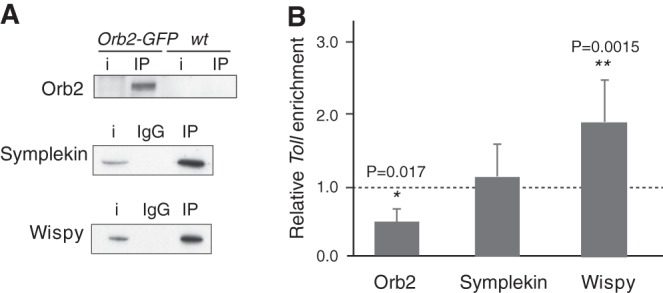
*Toll* mRNA interacts with Wispy. Interactions of *Toll* mRNA with various cytoplasmic polyadenylation factors was assessed by RIP from *Drosophila* 90 min embryo extracts. (*A*) Orb2-GFP was immunoprecipitated with GFP-binder beads from *Orb2-GFP*, or *OrR* (*wt*) embryos used as negative control. Symplekin and Wispy were immunoprecipitated from *OrR* embryos, carrying nonspecific IgG as negative control (IgG). Fifteen percent (Symplekin, Wispy) or 50% (Orb2-GFP) of the pellet was loaded to visualize protein; i, 3%–10% input extract. (*B*) The level of *Toll* mRNA in the pellet was measured by RT-qPCR, normalized to the negative control, and represented as enrichment over immunoprecipitation of the nonpolyadenylation substrate *sop* (dashed line). The plot represents the average of at least three independent experiments. Error bars were calculated as standard deviation, and statistical significance analyzed by unpaired Student's *t*-test (*) *P* < 0.05; (**) *P* < 0.01; (***) *P* < 0.001. See Supplemental Table S1 for raw data and normalization details.

To test whether homologs of known cytoplasmic polyadenylation factors could play a role in noncanonical polyadenylation of *Toll*, we tested their expression in early (90 min) embryos and analyzed their association to *Toll* mRNA. We focused on Orb2, Wispy, and Symplekin. The two latter factors are readily detected by western blot with specific antibodies (Fig. 1A, input). Because we lacked antibodies against Orb2, we used an Orb2-GFP *Drosophila* line generated by homologous recombination (Krüttner et al. 2012). Although expression of Orb2 in the early embryo was weak, the protein could be detected after immunoprecipitation (IP) (Fig. 1A, upper panel). We next measured the presence of *Toll* mRNA in the immunoprecipitates by RT-qPCR, using IgG (in the case of Wispy and Symplekin) or wild-type embryo extracts (in the case of Orb2-GFP) as negative controls. As an additional control, we tested the presence of *sop* mRNA, an abundant transcript that is not subject to cytoplasmic polyadenylation (Benoit et al. 2008), and expressed the data as enrichment relative to *sop*. The results showed that Wispy, but not Orb2 or Symplekin, was associated to *Toll* mRNA (Fig. 1B; see raw data and normalizations in Supplemental Table S1).

**FIGURE 2.**
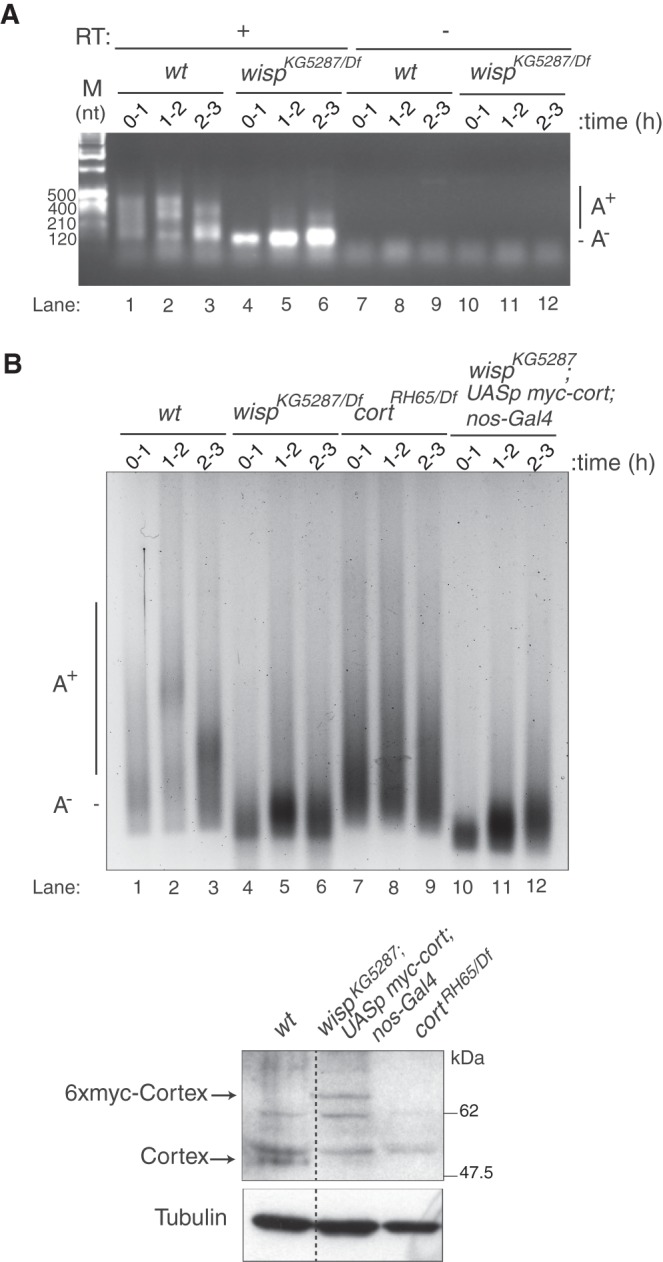
Wispy functions in polyadenylation of *Toll.* (*A*) *Toll* poly(A) tail length was assessed by PAT assay of embryos from *wt* and *wispy* mutant mothers collected at increasing times after egg laying. *wisp^KG5287^/Df* was *wisp^KG5287^/Df(1)RA47.* RT, reverse transcriptase. (*B*) As in *A* following the ePAT protocol. *cort^RH65^/Df* was *cort^RH65^/Df(2L)exel7027*. In *wisp^KG5287^; UASp-myc-cort/nos-Gal4*, a Cortex protein containing six myc tags (6xmyc-Cortex) was expressed in the ovarian germline using the *UASp-myc-cort* transgene and the *nos-Gal4* driver. The *bottom* panel shows a western blot of stage 14 oocytes to visualize Cortex. α-Tubulin was used as a loading control. Endogenous Cortex and myc-Cortex proteins were expressed at comparable levels. The dashed line indicates that lanes were separated in the original gel.

To address whether Wispy was involved in cytoplasmic polyadenylation of *Toll*, we measured the poly(A) tail length of *Toll* mRNA in embryos from Wispy mutant mothers using the PCR-based poly(A)-test (PAT) assay. *Toll* poly(A) tail was not elongated in the absence of Wispy (Fig. 2A, cf. lanes 1–3 with 4–6; Cui et al. 2008).

Embryos from *wispy* mutant mothers do not develop due to primary defects in meiotic progression (Benoit et al. 2008; Cui et al. 2008). A key Wispy target during meiosis is *cortex* mRNA, which encodes a component of the APC complex required for completion of the meiotic division (Benoit et al. 2008). To assess whether the effect of Wispy on *Toll* polyadenylation was direct and not due to the lack of Cortex protein, we measured the poly(A) tail length of *Toll* in *cortex* mutants, and in *wispy* mutants in which Cortex levels were rescued using a transgene. The results showed that *Toll* was polyadenylated in *cortex* mutants, while polyadenylation was not rescued in *wispy* mutants expressing Cortex (Fig. 2B, cf. lanes 7–9 with 10–12). These results identify Wispy as the poly(A) polymerase required for *Toll* mRNA cytoplasmic polyadenylation.

### Dicer-2 associates with cytoplasmic polyadenylation substrates

**FIGURE 3.**
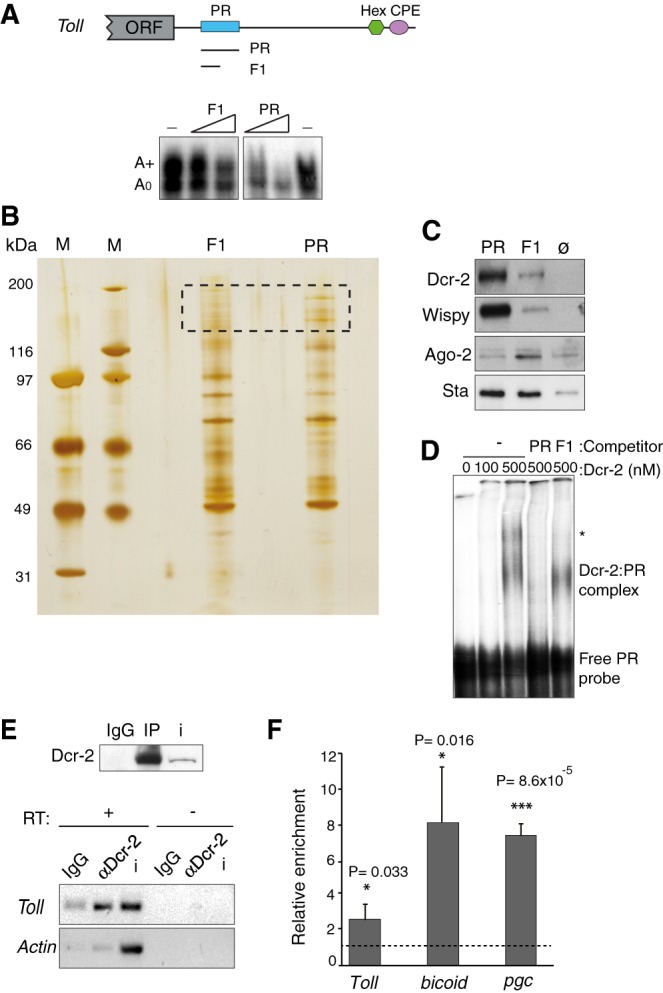
Dicer-2 is a potential noncanonical polyadenylation factor. (*A*) Schematic representation of *Toll* 3′ UTR depicting functional (PR) and nonfunctional (CPE, Hex) cytoplasmic polyadenylation elements. PR is a region of 180 nt that, when added in excess, competes polyadenylation of *Toll* (*lower* panel). F1 is an 86 nt fragment of PR that lacks competition capacity. Competitors were added at 100× and 300× molar excess over *Toll* RNA. (*B*) PR and F1 RNAs were used in RNA affinity chromatography, and eluates were separated by SDS–PAGE and silver stained (M, ladder). The region between 116 and 200 kDa (dashed square) was cut and analyzed by mass spectrometry. (*C*) RNA affinity chromatography followed by western blot confirms the recruitment of Dicer-2 and Wispy to PR, carrying Ago-2 detection as control. The ribosomal protein Stubarista (Sta) was used as loading control. Ø indicates chromatography performed with empty beads (no RNA). (*D*) Dicer-2 binds to PR directly. Recombinant Dicer-2 was incubated with radiolabeled PR at the indicated concentrations, and complexes were separated by native gel electrophoresis. Excess (300×) cold PR or F1 competitor were used to monitor specificity. The position of the free probe and complexes are indicated. The asterisk denotes a nonspecific complex. (*E*) Dicer-2 was immunoprecipitated from *OrR* 90 min embryo extracts (*upper* panel), and the presence of *Toll* and *actin* mRNAs in the pellet was tested by semi-quantitative RT-PCR (*bottom* panel). (RT) Reverse transcriptase. IP with nonspecific IgG was performed as negative control (IgG). i, 3% input extract. (*F*) Binding of Dicer-2 to the indicated mRNAs was assessed by RIP followed by RT-qPCR, as indicated in the legend of Figure 1B. Averages with the standard deviation of three independent experiments are represented. Statistical significance was analyzed by unpaired Student's *t*-test (*) *P* < 0.05; (**) *P* < 0.01; (***) *P* < 0.001. See Supplemental Table S1 for raw data and normalization details.

Contrary to nuclear poly(A) polymerases, Wispy lacks RNA-binding domains and associates with mRNA indirectly via RNA-binding proteins (for review, see Minasaki and Eckmann 2012). In the case of *Toll*, excess amounts of an RNA fragment including the polyadenylation signals (“polyadenylation region” or PR) competes polyadenylation, indicating that relevant *trans*-acting factors bind to this region (Fig. 3A; Coll et al. 2010). To identify these factors, we performed RNA affinity chromatography using PR as bait. As control, we used a fragment of PR with no competition capacity (F1, Fig. 3A). The results revealed high-molecular-weight factors binding to PR but not to F1 (Fig. 3B, square). These portions of the gel were subjected to proteomics analysis, revealing several PR-binding factors, most prominently Wispy and Dicer-2 (Supplemental Table S2). Independent chromatography experiments followed by western blot confirmed the association of Dicer-2 and Wispy to PR (Fig. 3C).

Binding of Wispy to PR is consistent with its role in *Toll* mRNA polyadenylation, and suggests that important *trans*-acting factors recruit Wispy to this region. Dicer-2 (Dcr-2) has been previously shown to promote the translation of *Toll* mRNA during immune signaling by binding to its 3′ UTR (Wang et al. 2015), suggesting that Dicer-2 could be the Wispy recruiting factor and that cytoplasmic polyadenylation may underlie the effects of Dicer-2 on translation. In support of this hypothesis, recombinant Dicer-2 binds to PR in electrophoretic mobility shift assays, yielding a complex that is competed by excess PR but not by F1 (Fig. 3D).

Dicer-2 is best known as an RNase III-like enzyme that functions in RNA interference. In *Drosophila*, there are two Dicer proteins, Dicer-1 and Dicer-2, which function in miRNA and siRNA processing, respectively (Lee et al. 2004). Dicer-2 processes dsRNA precursors into siRNAs, and subsequently associates with the double-stranded RNA-binding protein R2D2 to load siRNAs into Ago-2 leading to formation of siRISC, the effector RNAi complex (Marques et al. 2010). Importantly, Ago-2 did not bind to PR, suggesting RNAi-independent functions of Dicer-2 in cytoplasmic polyadenylation (Fig. 3C).

To test whether endogenous *Toll* mRNA associated with Dicer-2 in embryos, we performed RNA-immunoprecipitation (RIP) analysis. Dicer-2 was immunoprecipitated using an affinity-purified antibody, and the presence of *Toll* in the pellet was assessed by RT-PCR. Indeed, Dicer-2 specifically interacted with *Toll* mRNA (Fig. 3E). Follow-up RT-qPCR experiments indicated that Dicer-2 not only interacted with *Toll*, but also with other cytoplasmic polyadenylation substrates (*bicoid, pgc*) (Fig. 3F).

### Dicer-2 interacts with Wispy

**FIGURE 4.**
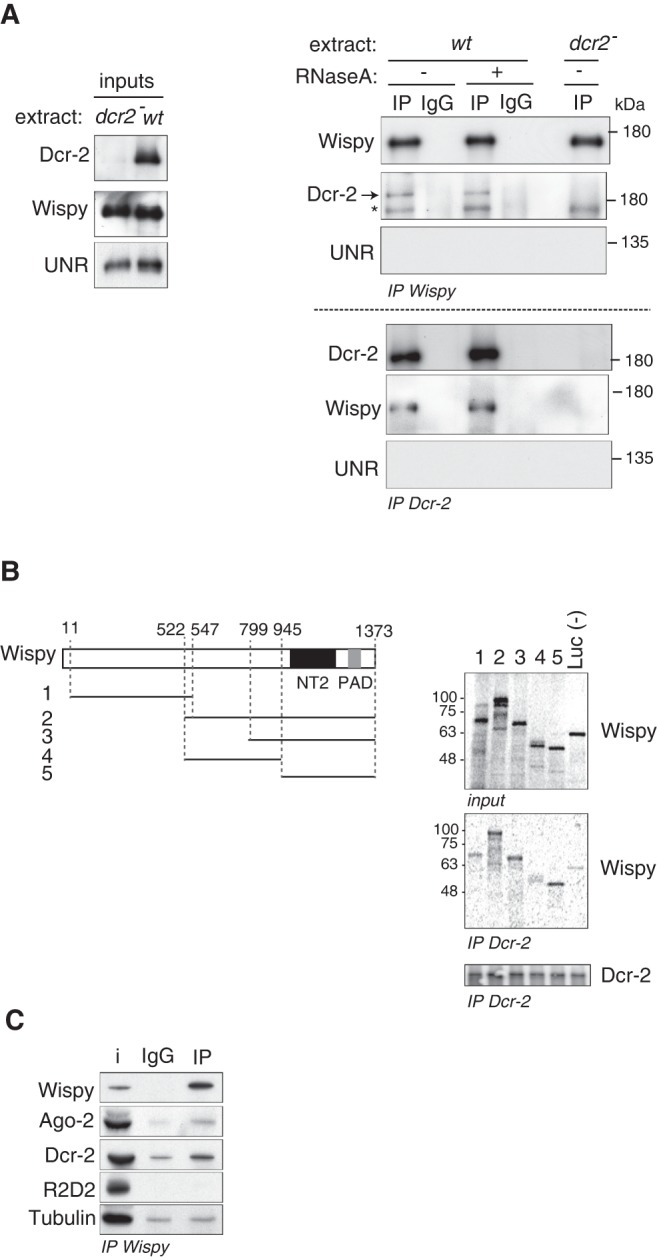
Dicer-2 interacts with Wispy. (*A*) Interaction of endogenous Wispy and Dicer-2 was assessed by coimmunoprecipitation from 90 min embryo extracts (*wt*). *dcr-2^L811fsx^* null embryo extracts (*dcr2^−^*) or immunoprecipitation with nonspecific IgGs were used as negative controls. The pellets were treated (+) or not (−) with 10 µg of RNase A. Inputs are shown on the *left*. UNR was included as a specificity control. The asterisk denotes a background band. (*B*) Dicer-2 interacts with the carboxy-terminal domain of Wispy. (*Left*) Schematic representation of Wispy and the fragments used in the experiment. (NT2) Nucleotidyl-transferase 2 domain; (PAD) poly(A) polymerase-associated domain. Numbers *above* Wispy indicate amino acid positions. (*Right*) Recombinant Dicer-2 was mixed with ^35^S-labeled Wispy fragments 1–5, and immunoprecipitated using affinity-purified antibodies. Firefly luciferase (Luc) was used as negative control. Wispy inputs (10%) and pellets (30%) are shown in the *upper* and *middle* panels, respectively. The *lower* panel shows the immunoprecipitated Dcr-2. (*C*) Ago-2, but not R2D2, interacts with Wispy. Immunoprecipitation of Wispy was performed as in *A*, and the presence of Ago-2, R2D2, and Dicer-2 in the pellet assessed by western blot. Tubulin was used as background control. i, 10% input.

We next tested whether Dicer-2 interacts with Wispy. We used extracts from wild type and *dicer-2^L811fsX^* null embryos as control (Lee et al. 2004). While these embryos lack Dicer-2, they contain equivalent amounts of Wispy (Fig. 4A, left panel). Immunoprecipitation of Wispy revealed a fraction of Dicer-2 that interacted with Wispy, results that were confirmed with the reverse immunoprecipitation (Fig. 4A, right panel). The interaction was specific, as a control abundant RNA-binding protein (UNR) was not coimmunoprecipitated. In addition, the interaction was resistant to digestion with RNase A, suggesting that the two proteins are not simply bridged by RNA. To confirm a direct interaction of Dicer-2 with Wispy, we tested the association of several Wispy fragments produced by in vitro translation with recombinant Dicer-2 using coimmunoprecipitation. The results showed that Wispy fragments containing the carboxy-terminal region, including the nucleotidyl-transferase (NT2) and PAP associated (PAD) domains, coimmunoprecipitated with Dicer-2 (Fig. 4B, fragments 2, 3, and 5), while the amino-terminal and central domains of Wispy (fragments 1 and 4) showed interaction levels similar to the negative control (Firefly luciferase, Luc). We conclude that Dicer-2 interacts with the carboxy-terminal domain of Wispy, in a likely direct manner.

To address whether the interaction of Wispy with Dicer-2 occurred in the context of RNAi complexes, we tested whether Wispy interacted with Ago-2 and R2D2 using coimmunoprecipitation in embryo extracts. Although Wispy was found to weakly interact with Ago-2, it did not interact with R2D2 (Fig. 4C). These data support an RNAi-independent function of the Wispy–Dicer-2 complex.

### Dicer-2 functions in cytoplasmic polyadenylation

**FIGURE 5.**
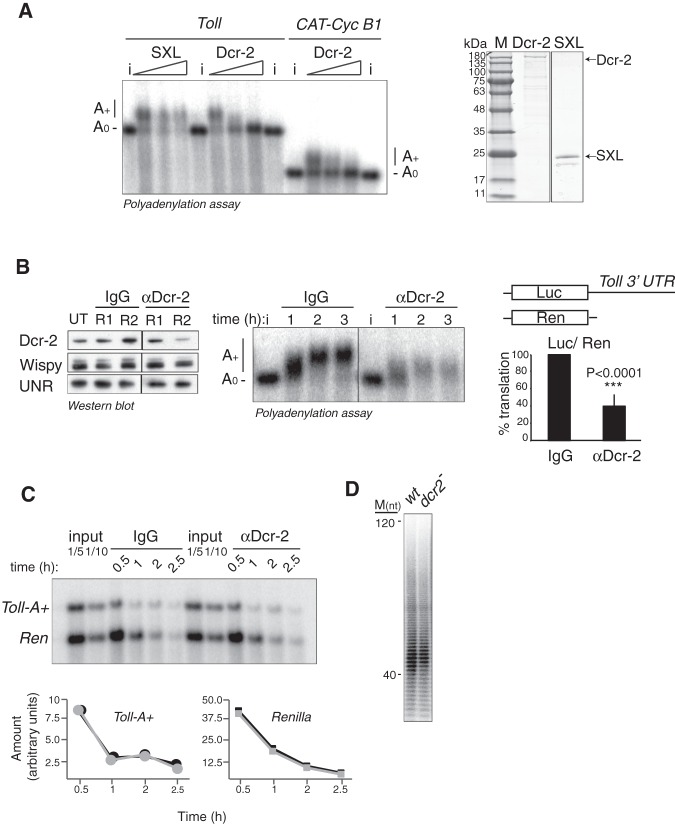
Dicer-2 functions in cytoplasmic polyadenylation. (*A*) Excess Dicer-2 squelches cytoplasmic polyadenylation. Polyadenylation of *Toll* and *CAT-Cyc B1* 3′ UTRs in the presence of increasing amounts of recombinant Dcr-2 (0, 100, and 200 nM). The unrelated protein SXL (amino acids 122–354) was used as negative control. i, 10% input. The quality of the proteins was assessed by colloidal-blue staining (*right* panel). (*B*) Depletion of Dicer-2 reduces *Toll* polyadenylation and translation. (*Left*) Efficiency of immunodepletion after one (R1) and two (R2) rounds of depletion. Dicer-2 depletion does not affect Wispy levels. UNR was used as loading control. (*Middle*) Polyadenylation of radiolabeled *Toll* 3′ UTR in Dicer-2- and mock (IgG)-depleted extracts. The polyadenylation profile at 1, 2, and 3 h of incubation is shown. (*Right*) Translational efficiency of Dicer-2- and IgG-depleted extracts. Translation of a *Firefly* luciferase reporter containing the *Toll* 3′ UTR was measured. *Renilla* luciferase mRNA was cotranslated as an internal control. Firefly luciferase was corrected for Renilla expression and referred to the activity in IgG-depleted extracts. Values represent the average ± STDEV from four independent experiments. Statistical significance was assessed by Student's *t*-test. (*C*) Depletion of Dicer-2 does not affect *Toll* deadenylation rate. Cordycepin-labeled *Toll-A+* and *Renilla* mRNAs were coincubated in IgG- and Dicer-2- depleted extracts, and samples collected at 30 min, 1, 2, and 2.5 h of the reaction. RNAs were visualized in a 1.5% denaturing agarose gel and quantified (*bottom*); black, mock-depleted extract; gray, Dcr-2-depleted extract. Two dilutions of the input were loaded as reference. (*D*) Dicer-2 does not affect global polyadenylation states. Assessment of global poly(A) tail lengths in *wt* and *dcr-2* null embryos.

To test whether Dicer-2 is a cytoplasmic polyadenylation factor, we first performed squelching assays. We added purified, recombinant Dicer-2 to *Drosophila* extracts and asked whether these could support cytoplasmic polyadenylation. We expected that, when added in excess, Dicer-2 squelches cytoplasmic polyadenylation factors (e.g., Wispy) and inhibits polyadenylation. We found that, indeed, excess Dcr-2 prevented polyadenylation of *Toll* and *Xenopus cyclin B1* (*CAT-CycB1*) mRNAs, while excess of a control protein (SXL) did not (Fig. 5A).

We next depleted Dcr-2 from extracts using affinity-purified antibodies. Typically, two to three rounds of depletion were necessary to reduce the amount of Dicer-2 (Fig. 5B, left panel). The amount of Wispy in depleted and control mock-depleted extracts was equivalent (Fig. 5B, left panel). We then tested polyadenylation of exogenously added radiolabeled *Toll* 3′ UTR in these extracts. We found that both polyadenylation and stability of *Toll* were affected (Fig. 5B, middle panel). Consistent with these defects, translation of a reporter containing the *Toll* 3′ UTR decreased in Dicer-2-depleted extracts (Fig. 5B, right panel). The stability defect is likely a direct consequence of reduced poly(A) tail length, as the poly(A) tail promotes not only translation but also mRNA stability (Barckmann and Simonelig 2013; Wahle and Winkler 2013). The reduced poly(A) tails of *Toll* in Dicer-2-depleted extracts are consistent with two possible scenarios: (i) Dicer-2 promotes cytoplasmic polyadenylation or, (ii) Dicer-2 inhibits deadenylation. To distinguish between these possibilities, we uncoupled polyadenylation from deadenylation using cordycepin (3′ deoxyadenosine). Addition of cordycepin to the 3′ end of a transcript prevents further adenylation but not deadenylation. We therefore added radiolabeled cordycepin to the 3′ end of a polyadenylated *Toll* transcript and monitored the fate of this mRNA in mock- and Dicer-2-depleted extracts. As an internal control, *Toll* was coincubated with cordycepin-labeled *Renilla* mRNA, which should not show changes upon Dicer-2 depletion. We reasoned that, if Dicer-2 inhibits deadenylation, the rate of cordycepin disappearance from *Toll* should be higher in Dicer-2-depleted extracts. However, we found equivalent cordycepin signals along time for mock- and Dicer-2-depleted extracts (Fig. 5C). We conclude that Dicer-2 behaves as a cytoplasmic polyadenylation factor. This function should be restricted to a specific population of mRNAs, because the bulk poly(A) length of mRNAs remains constant in *dicer-2* null embryos (Fig. 5D).

### Dicer-2 promotes the cytoplasmic polyadenylation of *r2d2* mRNA

**FIGURE 6.**
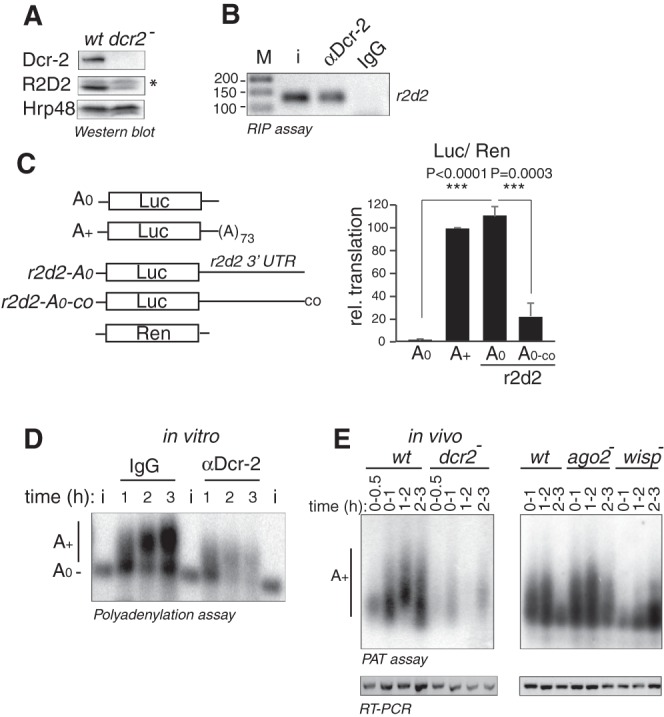
Dicer-2 promotes polyadenylation of *r2d2* mRNA. (*A*) R2D2 protein levels decrease in *dcr-2* null flies. Protein levels were assessed by western blot. Hrp48 was used as loading control. The asterisk denotes a nonspecific band. (*B*) Dicer-2 binds to endogenous *r2d2* mRNA. Dicer-2 was immunoprecipitated from 90 min embryo extracts and the presence of *r2d2* mRNA in the pellet assessed by RT-PCR. M, marker; i, input. (*C*) *r2d2* 3′ UTR promotes translation in a polyadenylation-dependent manner. The translation efficiency of a luciferase reporter containing the 3′ UTR of *r2d2* (*r2d2-A*_0_) was compared with reporters lacking *r2d2* and either containing (*A+*) or not (*A*_*0*_) a poly(A) tail. An *r2d2* construct with cordycepin at its 3′ end was included (*r2d2-A_0_-co*). Translation was assessed as described in the legend of Figure 5B. The plot represents the average ± STDEV of three experiments. Statistical significance was analyzed by unpaired Student's *t*-test. Cordycepin prevents translation of *r2d2*, suggesting that the reporter acquires a poly(A) tail during the reaction. (*D*) *r2d2* 3′ UTR is polyadenylated in embryo extracts, and Dicer-2 depletion reduces the polyadenylation efficiency. Assays were performed as described in the legend of Figure 2B. (*E*) Polyadenylation of *r2d2* mRNA is reduced in *dcr-2* and *wispy*, but not in *ago2* null embryos. PAT assays were performed in *wt* (*w*^*1118*^), *dcr-2^L811fsX^*, *wisp^KG5287/Df^*, and *ago2*^*414*^ embryos as indicated in the legend of Figure 2A. To increase the sensitivity of the assay in *dcr-2* extracts, amplified products were visualized by Southern blot using an *r2d2*-specific probe (*upper left* panel). To ensure that *r2d2* mRNA was present in null embryos, semi-quantitative PCR with oligonucleotides detecting *r2d2* was performed from the same RNA samples used for the PAT assay (*lower* panels).

It has been reported that *dicer-2* null embryos lack R2D2 (Fig. 6A; Liu et al. 2006; Lim et al. 2008). This defect has been attributed to Dicer-2 promoting the stability of R2D2 protein, although this possibility has not been directly tested (Liu et al. 2006). Ectopic R2D2 can be efficiently expressed in Dicer-2-depleted cells, indicating alternative regulatory mechanisms (Nishida et al. 2013). Given the role of Dicer-2 as a cytoplasmic polyadenylation factor, we tested whether Dicer-2 could promote the cytoplasmic polyadenylation and translation of *r2d2* mRNA. In agreement with an RNA-related function of this protein, Dicer-2 binds to *r2d2* mRNA in *Drosophila* embryos (Fig. 6B). In addition, a reporter containing the 3′ UTR of *r2d2* was translated at higher levels than a reporter without this UTR in vitro, and at levels similar to a polyadenylated reporter (Fig. 6C). Importantly, addition of cordycepin to the 3′ end of the *r2d2* reporter inhibited translation, indicating that translation of *r2d2* mRNA is driven by cytoplasmic polyadenylation (Fig. 6C). Indeed, the *r2d2* 3′ UTR was polyadenylated in embryo extracts, and this polyadenylation dramatically decreased in the absence of Dicer-2 (Fig. 6D). Consistent with these in vitro results, endogenous *r2d2* mRNA was polyadenylated in vivo, and the extent of polyadenylation decreased in *dcr-2* or *wispy* null embryos, while it remained unaffected in *ago2* null embryos (Fig. 6E). These results indicate that Dicer-2 regulates *r2d2* expression at the level of cytoplasmic polyadenylation in an RNAi-independent manner.

## DISCUSSION

The mechanisms governing cytoplasmic polyadenylation in early embryos are poorly understood. We have previously shown evidence for a noncanonical machinery that functions independently of the CPE and the Hex in *Drosophila* embryos (Coll et al. 2010). Here we identify Dicer-2 and Wispy as components of this machinery. These results uncover a novel function of Dicer-2 in mRNA activation through cytoplasmic polyadenylation.

Cytoplasmic poly(A) polymerases are thought to be recruited to the 3′ UTRs of substrate mRNAs by RNA-binding factors. This is best illustrated in *C*. *elegans*, where the polymerase GLD-2 can be recruited to the mRNA by GLD-3, RNP-8 or other RNA-binding proteins, contributing to a major mechanism to control cell identity during early embryogenesis (Kim et al. 2009; Elewa et al. 2015). During *Xenopus* oocyte maturation, GLD-2 is recruited to the mRNA by the concerted action of CPEB, CPSF, and Symplekin (Barnard et al. 2004). Alternative proteins for recruitment of cytoplasmic poly(A) polymerases and/or efficient polyadenylation have been described, including αCP2 in *Xenopus* embryos or QKI-7 in somatic mammalian cells (Vishnu et al. 2011; Yamagishi et al. 2016). However, these factors rely on the Hex—and by inference on CPSF—for efficient polyadenylation. The noncanonical machinery that operates in *Drosophila* embryos is different in that it is totally independent of the classical polyadenylation elements (Coll et al. 2010). This machinery contains Dicer-2, a Wispy-binding factor (Figs. 3, 4). The Dicer-2 polyadenylation machinery is important to achieve appropriate levels of Toll and R2D2 during early development (Figs. 5, 6; Coll et al. 2010). As R2D2 cooperates with Dicer-2 during RNAi, stimulation of *r2d2* mRNA translation by Dicer-2 may enforce RNAi in the early embryo.

Three lines of evidence are consistent with an RNAi-independent function of Dicer-2 in cytoplasmic polyadenylation. First, Dicer-2 associates with PR in the absence of Ago-2 (Fig. 3; Supplemental Table S2). Second, Wispy interacts with Dicer-2, but not with R2D2 (Fig. 4). Third, we detect no processing of *Toll* or *r2d2* mRNAs during cytoplasmic polyadenylation (data not shown). Binding of *Drosophila* Dicer-2 to these transcripts without apparent processing is consistent with previous findings showing that human and *C. elegans* Dicer can bind to mRNA in a “passive” manner (Rybak-Wolf et al. 2014). Passive sites are characterized by stem–loops with short stems, where Dicer binding is detected at the loop region. We have performed SHAPE structural analysis of the *Toll* PR region and indeed find three such stem–loops (Supplemental Fig. 1). Binding of recombinant Dicer-2 to PR occurs at high protein concentrations (500 nM), suggesting that other proteins or additional *Toll* RNA elements contribute to efficient Dicer-2 binding to this region. The proteins in Supplemental Table S2 represent potential candidates. Furthermore, consistent with shared polyadenylation factors which are distinct from those operating in the canonical process, *Toll* polyadenylation is competed by excess of *r2d2* RNA and vice versa, while polyadenylation of none of these transcripts is competed by *CycB1* RNA (Supplemental Fig. 2).

A previous report showed that Dicer-2 and Ago-2 can regulate poly(A) tail lengths in *Drosophila* S2 cells (Siomi et al. 2005). Our results differ from this report in several important aspects. First, poly(A) tail regulation in S2 cells was linked to the RNAi machinery and to the capacity of Dicer-2 to prevent deadenylation by the CCR4/NOT/CAF1 complex. We show here that, in embryos, Dicer-2 regulates cytoplasmic polyadenylation in an RNAi- and deadenylation-independent fashion. Second, Siomi found that poly(A) tail shortening upon Dicer-2 depletion correlates with increased protein levels; however, we find a translation-promoting role of Dicer-2. Third, in S2 cells Dicer-2 maintained the poly(A) tail of a reporter EGFP transgene without apparent specificity signals, but did not affect the poly(A) tail of a transiently expressed reporter, and effects on endogenous transcripts were not reported. We find that, in embryos, Dicer-2 functions on both endogenous and exogenous transcripts containing signals for cytoplasmic polyadenylation. Finally, we have found that SL2 cells express no Wispy and rather low levels of other cytoplasmic poly(A) polymerases (Supplemental Fig. 3). Altogether, these data suggest that different phenomena are observed in cultured cells and embryos.

Our data are in agreement with recent findings of an RNAi-independent function of Dicer-2 in Toll immune signaling, where Dicer-2 was found to increase the translation of *Toll* mRNA and promote resistance to fungal infection (Wang et al. 2015). We provide here a molecular mechanism for translational control by Dicer-2 in embryos. In addition to the reported increased sensitivity to infection, we find that the loss of Dicer-2 causes a dramatic reduction in fertility: Males are sterile while females lay 18% eggs compared with wild-type flies (Table 1). These data suggest that, in addition to *Toll* and *r2d2*, Dicer-2 might be important for the expression of other transcripts. Furthermore, *dcr-2* mutants have been shown to exhibit reduced life-span and hypersensitivity to a whole array of stress conditions including oxidative, endoplasmic reticulum, starvation and cold stress (Lim et al. 2011). Intriguingly, reductions—rather than increases—in protein and mRNA abundance were most often detected under these conditions, raising the possibility that the mechanism we describe here for Dicer-2 in mRNA activation can be extended to adult flies.

**TABLE 1.**
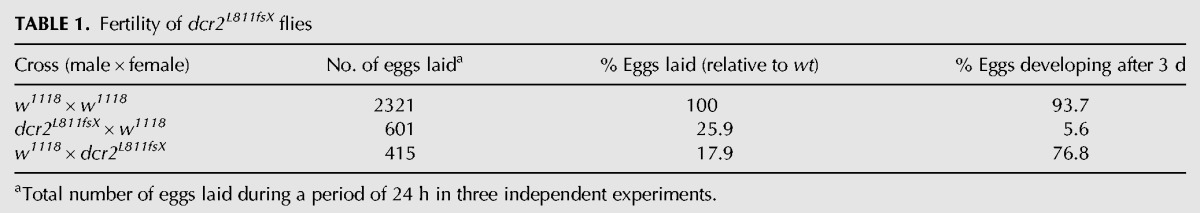
Fertility of *dcr2*^*L811fsX*^ flies

Very recently, another small RNA factor was reported to interact with Wispy. Aubergine, an Argonaute protein of the Piwi-interacting RNA pathway, interacts with a central region of Wispy and promotes cytoplasmic polyadenylation and stabilization of mRNAs involved in germ cell specification and development at the posterior pole of the embryo (Dufourt et al. 2017). This highlights the interconnection between small RNA factors and the cytoplasmic polyadenylation machinery.

In addition to Wispy, other poly(A) polymerases are expressed in early embryos (Supplemental Fig. 3). Together with the variety of RNA-binding factors, this increases the diversity of potential polyadenylation machineries exponentially. Identifying these machineries, their conservation, their substrates and relevance in development and cell homeostasis are important questions for future research.

## MATERIALS AND METHODS

### Flies

*OrR* and *w* ^*1118*^ were used as wild-type flies. The following mutant or transgenic stocks were used: *Orb2-GFP* (kindly provided by Dr. Krystyna Keleman), *w; Dcr2^L811fsX^/CyO, amos^Roi-1^*, *wisp*^*KG5287*^ (a *wisp* null allele) and *Df(1)RA47* that overlaps *wisp* (Benoit et al. 2008), *cort*^*RH65*^ and *UASp-myc-cortex* (Pesin and Orr-Weaver 2007), *Df(2L)exel7027* that overlaps the *cortex* gene (Bloomington Stock Center), *ago2*^*414*^ (Okamura et al. 2004), and *nos-Gal4* (Rørth 1998). Flies were maintained on standard food at 25°C.

To produce *Dicer-2* null embryo extracts, *w ;Dcr2^L811fsX^* homozygous females were crossed with *w*^*1118*^ males and embryos were collected at 90 min after egg laying. To ensure uniform staging of embryos, trays were exchanged three times before the final embryo collection. As a control, the same procedure using *w*^*1118*^ females was followed. *wisp* and *cortex* mutant females were also crossed with *w*^*1118*^ males.

### Protein expression

Dcr-2 was expressed in Baculovirus-infected Sf9 cells from a pFastBac-HisDcr-2His construct kindly provided by Dr. Quinghua Liu, following the recommendations of the Bac-to-Bac Baculovirus Expression System Manual (Invitrogen) for expression, and the protocol described by Ye and Liu (2008) for purification.

SXL (amino acids 122-354) was expressed in *E. coli* as a His-tagged fusion and purified as previously described (Hennig et al. 2014). Briefly, SXL expression was induced with 1 mM IPTG for 3 h at 37°C, and cell pellets were resuspended in lysis buffer [500 mM NaCl, 50 mM NaH_2_(PO)_4_ pH 8.0, 10 mM imidazole, 1× Roche protease inhibitors mix]. Cells were sheared, centrifuged, and the SXL protein in the supernatant purified using Ni-NTA columns. All proteins were dialyzed against buffer D (20 mM HEPES pH 8.0, 20% glycerol, 1 mM DTT, 0.01% NP-40, 0.2 mM EDTA).

Wispy fragments were expressed as ^35^S-labeled proteins in RRL and TNT in vitro translation systems following the instructions from the manufacturer.

### Antibodies

Antibodies against Dicer-2 and R2D2 were originally provided by Dr. Qinghua Liu (Liu et al. 2003). We also raised anti-Dicer-2 polyclonal antibodies against the first 151 aa of Dicer-2 in rabbits. These antibodies were affinity-purified in a HiTrap NHS-activated HP column (GE Healthcare) coupled with His-Dicer-2(1-257)-MBP protein. Antibodies against aa 1–300 of Ago2 and full-length stubarista (Sta) were generated in rabbits. Antibodies against Symplekin (Sullivan et al. 2009), Wispy (Cui et al. 2008), and Cortex (Pesin and Orr-Weaver 2007) were kindly provided by Drs. Bill Marzluff, Mariana Wolfner, and Terry Orr-Weaver, respectively. Anti-UNR antibodies were previously described (Abaza et al. 2006). Anti-GFP (Invitrogen, A6455) and mouse anti-αTubulin (T5168, Sigma) were provided commercially. These antibodies were used in western blot at the following dilutions: anti-Dcr-2 1:500 and 1:2000, anti-Ago-2 1:1000, anti-Sta 1:2000, anti-Symplekin 1:1000, anti-Wispy 1:500 and 1:2000, anti-GFP 1:1000, anti-Cortex 1:2000, anti-UNR 1:2000, anti-Hrp48 1:10,000, anti-αTubulin 1:20,000.

### Plasmids

DNA constructs for the generation of *Toll* 3′ UTR, *Luc-Toll*, *Luc-A+, Luc-A_0_*, and *CAT-CycB1* mRNAs were previously described (de Moor and Richter 1999; Coll et al. 2010). To generate the *r2d2* reporter, the 3′ UTR of *r2d2* mRNA was amplified by RT-PCR from 90 min embryo extracts using oligonucleotides r2d2-f-SmaI (5′-TCCCCCGGGTGCGTATAACATTTATTCAAC) and r2d2-r-SalI (5′-ACGCGTCGACTTTATTTATTTTAGACTGAAA), and cloned into the SmaI/SalI sites of pBSK. This fragment was also cloned downstream from the Firefly luciferase ORF in pBSK to obtain r2d2-A_0_. Wispy fragments 1–3 (Fig. 4B) were cloned into pCSH3 (Joly et al. 2013), and fragments 4 and 5 into pGEMT. The fragments were engineered to contain HA-tags at the amino terminus. Fragment 5, in addition, contains a Flag-tag at the C terminus. All constructs were verified by sequencing.

### In vitro transcription

mRNAs were synthesized as described previously (Gebauer et al. 1999). RNAs either lacked or contained a poly(A) tail of 73 residues, as indicated. mRNAs used for translation contained a ^7m^GpppG cap, and RNAs used for competition contained an ApppG cap. RNAs used for polyadenylation were trace-labeled with α^32^P-UTP and contained an ^7m^GpppG cap.

Biotinylated transcripts used in affinity chromatography assays were synthesized using the Megashort Script kit (Invitrogen) following the manufacturer's instructions. In these reactions, bio-CTP was added at a ratio of 1:1 compared with CTP. These RNAs contained an ApppG cap. The quality and quantity of all RNAs were monitored by visualization in agarose gels.

### Labeling with cordycepin

Labeling with radioactive cordycepin was used to determine global poly(A) tail lengths, or to prevent polyadenylation of reporters. Labeling was performed with yeast PAP (kindly provided by Drs. Elmar Wahle and Uwe Kühn) as follows. Total RNA from *Drosophila* 90 min embryos (1.5 µg) or in vitro synthesized *Toll-A+* and *Renilla* mRNAs (0.03 pmol) were incubated with 200 ng yeast PAP in a reaction of 20 µL containing 20 mM TRIS-HCl pH 7.0, 50 mM KCl, 10% glycerol, 1 mM DTT, 0.7 mM Mn-II-Cl_2_, 40 U RNasin and 2 µL ^32^αP-cordycepin (PerkinElmer NEG026250UC). After 30 min incubation at 30°C, 10 µg yeast tRNA was added as carrier, and RNAs were treated with phenol-chloroform and precipitated.

For determination of global poly(A) tail length, 10^5^ cpm were treated with 2 µg RNase A and 50 U RNase T1 in a final volume of 80 µL containing 200 mM NaCl and 10 mM TRIS-HCl pH 8.0, for 45 min at 30°C. The reaction was stopped by adding 20 µL of a mix containing 10 µg proteinase K, 5 µg glycogen, 5% SDS and 50 mM EDTA, and incubating for 30 min at 37°C. RNAs were then precipitated (without phenol extraction) and separated in a 12% acrylamide-urea sequencing gel.

### Cytoplasmic polyadenylation and translation assays

Cytoplasmic extracts from staged 90 min embryos were obtained as previously described (Gebauer et al. 1999). Polyadenylation and translation assays in these extracts were performed as previously described, without the addition of tRNA (Gebauer et al. 1999). Typically, 0.01 pmol of substrate mRNA were used per 12.5 µL reaction. In translation reactions, *Renilla* mRNA was cotranslated as an internal control. Luciferase activity was measured using the Dual Luciferase Assay System (Promega), and Firefly luciferase values were corrected for *Renilla* expression. In polyadenylation reactions, radiolabeled RNAs were used and visualized by separation in 1% denaturing agarose gels.

### PAT assays

PAT assays were performed as previously described (Coll et al. 2010), or using the ePAT method (Jänicke et al. 2012) for Figure 2B. Oligos for amplification of *Toll* and *r2d2* poly(A) tails were: PAT-r2d2-f 5′TTTGCCTTTAAAAGGAATGCTAGTAATC3′; PAT-Toll-f 5′GGTACCTACAGATTTATGCAGAC3′; and oligo(dT)-anchor 5′GCGAGCTCCGCGGCCGCGTTTTTTTTTTTT3′. To increase the sensitivity and test the specificity of *r2d2* PAT assays, PAT products were subjected to Southern blot analysis using a *r2d2*-specific probe. To ensure that null embryos contained *r2d2* mRNA, *r2d2* was amplified by semi-quantitative PCR from the same RNA samples used in PAT assays.

### Dicer-2 immunodepletion

Dicer-2 was immunodepleted from 1 mg embryo extract by two consecutive incubations with 100 µL protein A Dynabeads containing affinity purified anti-Dicer-2 antibody. Briefly, 100 µL beads were washed three times with five volumes of wash buffer (10 mM HEPES pH 8.0, 8% glycerol) and blocked with 1 mg embryo extract for 1 h at room temperature. Beads were then washed (4 × 5 vol wash buffer) and 20 µg of affinity-purified anti-Dicer-2 antibody or rabbit IgG were added. The suspension was incubated for 1 h at room temperature, and the beads were washed (5 × 5 vol wash buffer) and all liquid removed. One milligram of embryo extract were then added, and the mix incubated on ice for 30 min with occasional stirring. The extract was collected, added to fresh beads, and the process repeated. Depletion was confirmed by western blotting.

### Immunoprecipitation (IP) and RNA immunoprecipitation (RIP)

For standard IP, 150 µg of *Drosophila* 90 min embryo extract were incubated with 25 μL of Protein A Dynabeads (Invitrogen) bound to the appropriate antibody in a final volume of 100 µL of 1× NET buffer (50 mM TRIS-HCl pH 7.5, 150 mM NaCl, 0.1% NP40, 1 mM EDTA). After 1 h of incubation at 4°C, beads were washed twice with 10 bead volumes of 1× NET, and treated or not with 10 µg RNase A for 30 min at room temperature. Beads were then washed with 10 vol 1× NET and the proteins extracted with SDS loading buffer.

For RIP, 500 µg of 90 min embryo extract were used in conditions similar to IP, except that the incubation was carried at 25°C, and five washes were performed with 5× bead volumes of 1× NET buffer. Proteins recovered from 15% of the beads were resolved by SDS–PAGE and 85% of the beads were used for RNA extraction. For Orb2-GFP RIP, 5 mg of embryo extract were incubated with 30 μL GFP-Trap_M beads (Chromotek) for 2 h at 4°C and the immunoprecipitation performed following the manufacturer's instructions; half of the sample was used to visualize protein by SDS–PAGE, while the remaining half was used for RNA extraction. RNA was extracted from the beads using TRIzol (Invitrogen) after addition of 5 µg tRNA as carrier. RNA from input samples was processed in parallel, and its quality assessed in agarose gels.

### RT-qPCR and semi-quantitative PCR

RNA was treated with Turbo DNase (Ambion) in a final volume of 25 µL. Five microliters were then used for reverse-transcription with SuperScript II (Invitrogen) using random primers and oligo(dT) following the instructions of the manufacturer. Similar reactions were assembled without Superscript II as negative controls. cDNA was amplified by either semi-quantitative PCR with Taq DNA polymerase (PCR Master Mix Promega) or qPCR with SYBR Green (Applied Biosystems). Typically, 1 µL of a (1:3) dilution of the cDNA was used for qPCR while 5 µL were used for semi-quantitative PCR. The quality and sequences of oligonucleotides used for amplification of *Toll*, *sop, bicoid,* and *pgc* are detailed in Supplemental Table S1. Oligos for qPCR of *r2d2* are as follows: r2d2-f 5′AATGCTGGCCTTAATCTCCA3′ and r2d2-r 5′TGCCTCCAATTCCTCCATAG3′.

### RNA affinity chromatography

Thirty microliters of streptavidin Dyneabeads (Life Technologies) were equilibrated in TCE buffer (16.8 mM creatine phosphate, 0.08 μg/μL creatine kinase, 24 mM HEPES pH 7.5, 0.6 mM magnesium acetate, and 80 mM potassium acetate), and blocked for 5 min with 100 ng/μL tRNA at room temperature. Thirty three picomoles of biotinylated RNA and 10 U RNasin were added to the beads and incubated under rotation for 1 h at 4°C. The supernatant was removed and 2 mg *Drosophila* embryo extract (previously clarified by centrifugation at 13.000 rpm for 15 min at 4°C) were added, together with 320 ng/μL heparin and protease inhibitors (Roche). The mix was incubated for 30 min at room temperature under rotation. Beads were then washed five times with 1 volume of TCE supplemented with 0.1% Triton X-100, 320 ng/μL heparin and 10% glycerol. RNA-bound proteins were eluted from the beads by incubation with 20 μL of elution buffer (10 mM Tris-HCl pH 7.2, 1 mM MgCl_2_, 40 mM NaCl, 2 µL RNase cocktail from Ambion AM2286) for 30 min at 37°C. Samples were separated on precasted 4%–12% gradient gels (Invitrogen) and silver-stained. Bands of interest were sliced from the gel and identified by mass spectrometry.

### Mass spectrometry

Gel bands were destained and subsequently digested with 0.1 µg trypsin overnight at 37°C. After digestion, peptides were extracted, acidified with formic acid and desalted with Empore C18 columns prior to LC–MS/MS analysis. Samples were analyzed using a LTQ-Orbitrap XL mass spectrometer (Thermo Fisher Scientific) coupled to an Agilent Technologies 1200 Series. All data were acquired with Xcalibur software v2.2. The Proteome Discoverer software suite (v1.3.0.339, Thermo Fisher Scientific) and the Mascot search engine (v2.3, Matrix Science) were used for peptide identification. The data were searched against an in-house generated database containing all *Drosophila* proteins from NCBInr database (# entries 215.956). A precursor ion mass tolerance of 7 ppm at the MS1 level was used, and up to three miscleavages for trypsin were allowed. The fragment ion mass tolerance was set to 0.5 Da. Oxidation of methionine and protein acetylation at the N terminal were set as variable modifications. Peptides have been filtered using a Mascot Ion Score of 20.

### Gel mobility shift assay (EMSA)

EMSA was performed as previously described (Abaza et al. 2006). Briefly, ^32^P-labeled PR was incubated with recombinant Dicer-2 in a final volume of 20 µL containing 3 µg tRNA, 100 mM KCl, 20 mM HEPES pH 8.0, 20% glycerol, 1 mM DTT, 0.01% NP-40, and 0.2 mM EDTA. Where indicated, competitor cold PR or F1 RNAs were added at 300× molar excess. After 30 min incubation on ice, complexes were separated in a native 4% acrylamide gel.

### SHAPE

Secondary RNA structure was determined using selective 2′-hydroxyl-acylation analyzed by primer extension (SHAPE) (Wilkinson et al. 2006). Primer extension reactions were assembled with RNA templates previously treated with NMIA (13 mM or 26 mM NMIA for PR and *Toll*3′ UTR, respectively, treated for 45 min at 37°C) or DMSO as negative control. Oligonucleotides complementary to different regions of PR were 5′-radiolabeled, hybridized with the template RNA, and extended using Superscript III retrotranscriptase (Sigma-Aldrich) using the manufacturer indications. The oligonucleotides were as follows: 5′-CAGATAACACTTAAAACAGAGTGAT and 5′-GCTTGCAATAATCCTAATGTAC. Samples were loaded in a 7% acrylamide/urea denaturing gel, dried and visualized in a PhosphorImager. A ladder consisting of a sequencing reaction using the same oligos on the plasmids from which the template RNAs were synthesized, was loaded in parallel to locate the positions of the NIMIA-reactive nucleotides. These sequencing reactions were performed using the Thermo Sequenase Cycle Seq kit (USB). Bands were quantified and the data fed into RNA fold to obtain a secondary structure model.

## SUPPLEMENTAL MATERIAL

Supplemental material is available for this article.

## Supplementary Material

Supplemental Material
