# Supplementary material for: Dicer-2 promotes mRNA activation through cytoplasmic polyadenylation
Source: RNA. 2018 Apr;24(4):529–39. doi: 10.1261/rna.065417.117 (PMC5855953; doi:10.1261/rna.065417.117)
Supplement: Supplemental Material [file supp_24_4_529__index.html]

Dicer-2 promotes mRNA activation through cytoplasmic polyadenylation — Supplemental Material 

# Dicer-2 promotes mRNA activation through cytoplasmic polyadenylation

## Supplemental Material

- Supplemental\_Fig\_S1.pdf
- Supplemental\_Fig\_S2.pdf
- Supplemental\_Fig\_S3.pdf
- Supplemental\_Table\_S1.xlsx
- Supplemental\_Table\_S2.xls
