# Supplementary material for: Dicer-2 promotes mRNA activation through cytoplasmic polyadenylation
Source: RNA. 2018 Apr;24(4):529–39. doi: 10.1261/rna.065417.117 (PMC5855953; doi:10.1261/rna.065417.117)
Supplement: Supplemental Material [file supp_065417.117_Supplemental_Fig_S1.pdf]

Coll_Fig S1

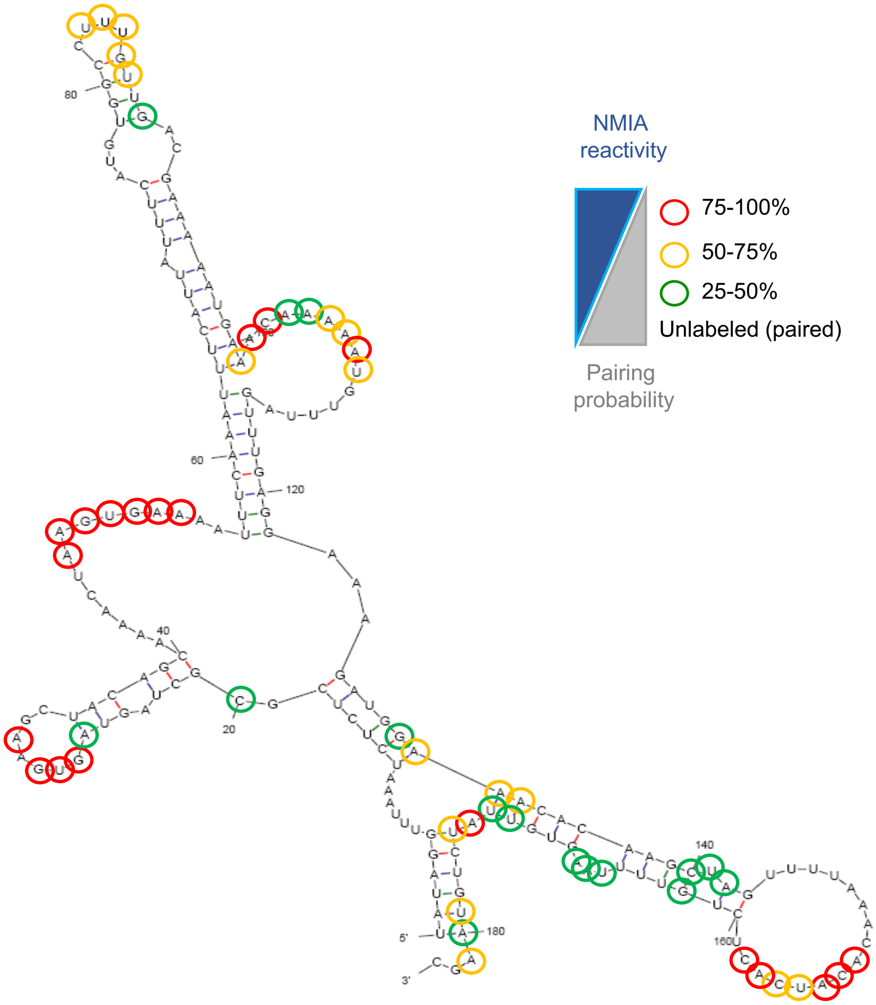

Structural model of PR. The secondary structure of PR was assessed by SHAPE of Toll 3’ UTR using oligonucleotides reading through the PR region. The reactivity to NIMIA is directly proportional to the probability of the residue to be in a single stranded conformation. Reactivity was classified into high (red), medium (yellow), low (green) or not reactive (unlabeled). The data were fed into RNA fold to yield a secondary structure model of PR.
