# Supplementary material for: Dicer-2 promotes mRNA activation through cytoplasmic polyadenylation
Source: RNA. 2018 Apr;24(4):529–39. doi: 10.1261/rna.065417.117 (PMC5855953; doi:10.1261/rna.065417.117)
Supplement: Supplemental Material [file supp_065417.117_Supplemental_Fig_S2.pdf]

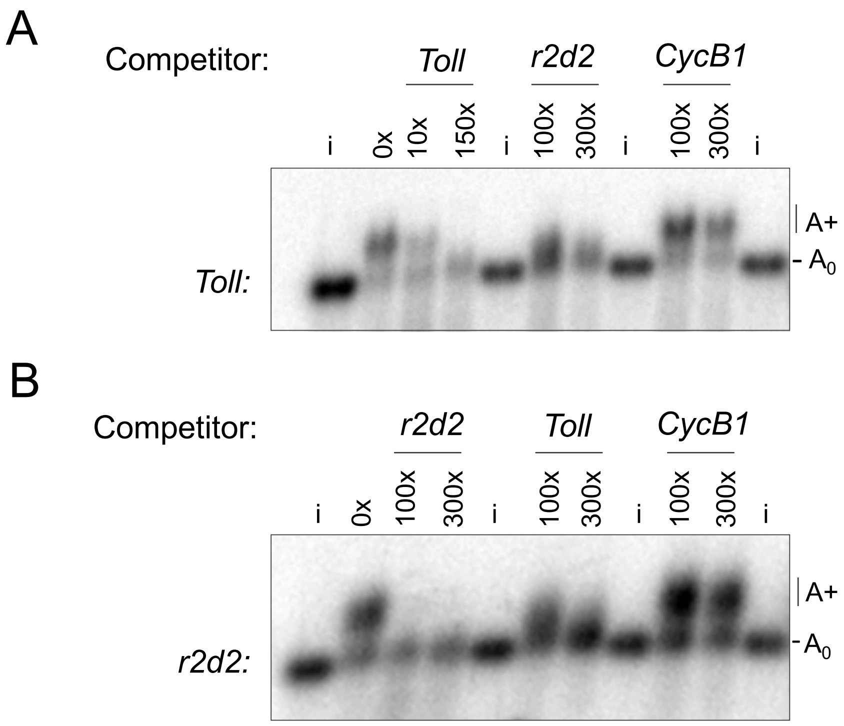

Toll and *r2d2* mRNAs share non-canonical polyadenylation factors. Polyadenylation competition assays of *Toll* (A) and *r2d2* (B) 3’ UTRs with the indicated molar excess of cold competitor RNAs. Cyclin B1 (CycB1) is a typical vertebrate polyadenylation substrate containing three CPEs and one hexanucleotide. i, input.
