# Supplementary material for: Dicer-2 promotes mRNA activation through cytoplasmic polyadenylation
Source: RNA. 2018 Apr;24(4):529–39. doi: 10.1261/rna.065417.117 (PMC5855953; doi:10.1261/rna.065417.117)
Supplement: Supplemental Material [file supp_065417.117_Supplemental_Fig_S3.pdf]

Coll_Fig S3

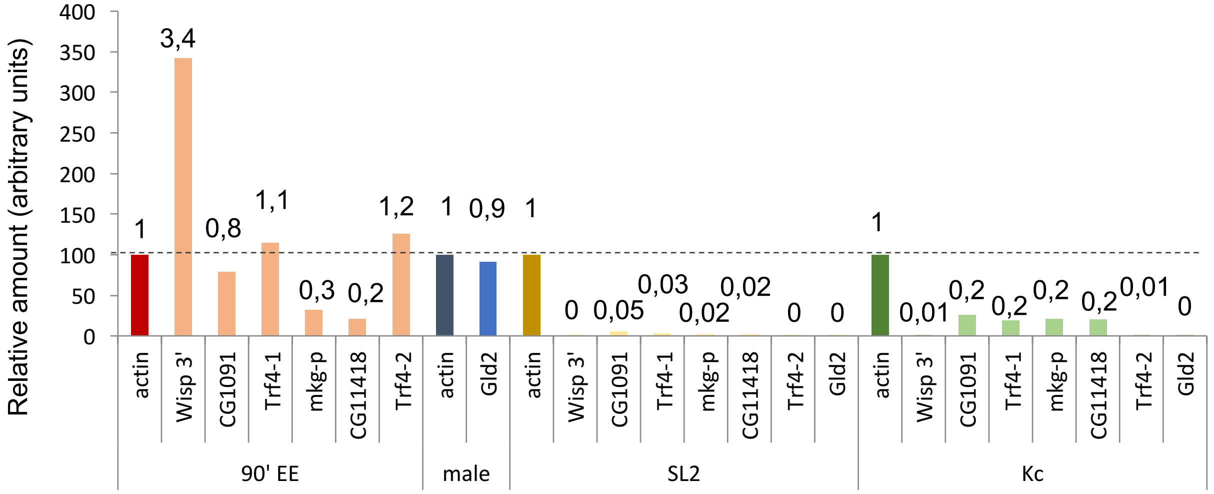

Expression of cytoplasmic poly(A) polymerases in *Drosophila* embryos and cultured cells. The levels of mRNAs encoding different cytoplasmic poly(A) polymerases in 90 min embryo extracts (90’ EE), SL2 and Kc cells were assessed by RT-qPCR, and the data represented relative to actin mRNA levels. Extracts from adult males were used as control for Gld-2. Numbers on top of each bar indicate the amount relative to actin.
